# Non-benzoquinone geldanamycin analogs trigger various forms of death in human breast cancer cells

**DOI:** 10.1186/s13046-016-0428-6

**Published:** 2016-09-22

**Authors:** Zhirui Zhang, Hong-Mei Li, Can Zhou, Qixiang Li, Linyan Ma, Zixuan Zhang, Yiming Sun, Lirong Wang, Xudong Zhang, Bing Zhu, Young-Soo Hong, Cheng-Zhu Wu, Hao Liu

**Affiliations:** 1 Faculty of Pharmacy, Bengbu Medical College, Bengbu, 233000 Anhui People’s Republic of China; 2 Department of Clinical medicine, Bengbu Medical College, Bengbu, 233000 Anhui People’s Republic of China; 3 Department of Pharmaceutical Sciences, School of Pharmacy, Computational Chemical Genomics Screening Center, Pittsburgh, PA USA; 4 School of Medicine and Public Health, University of Newcastle, Newcastle, NSW Australia; 5 Department of Gastrointestinal Surgery, The first Affiliated Hospital of Bengbu Medical College, Bengbu, 233000 Anhui People’s Republic of China; 6 Chemical Biology Research Center, KRIBB, Cheongju, 28116 Republic of Korea

**Keywords:** Geldanamycin analogs, Hsp90, Necroptosis, Apoptosis, Breast cancer

## Abstract

**Background:**

Hsp90 proteins are important therapeutic targets for many anti-cancer drugs in clinical trials. Geldanamycin (GA) was identified as the first natural inhibitor of Hsp90, increasing evidence suggests that GA was not a good choice for clinical trials. In this study, we investigated two new non-benzoquinone geldanamycin analogs of Hsp90 inhibitors, DHQ3 and 17-demethoxy-reblastatin (17-DR), to explore the molecular mechanisms of their anti-cancer activity in vivo and vitro.

**Methods:**

MTT and colony formation assays were used to measure cell viability. Flow cytometry, DAPI staining, ATP assay, electron microscopy, western blots, siRNAs transfection and immunofluorescence were used to determine the molecular mechanism of DHQ3- or 17-DR-induced different forms of death in human breast cancer MDA-MB-231 cells. Malachite green reagent was used to measure ATPase activity of the analogs.

**Results:**

DHQ3 and 17-DR presented efficiently inhibitory effect in MDA-MB-231 cell lines, and DHQ3 induced necroptosis by activation of the RIP1-RIP3-MLKL necroptosis cascade. And DHQ3-induced cell death was inhibited by a necroptosis inhibitor, necrostatin-1 (Nec-1), but not by a caspase inhibitor z-VAD-fmk. On the other hand, 17-DR induced apoptosis in MDA-MB-231 cells, indicating a caspase-dependent killing mechanism. We further demonstrated that down-regulation of RIP1 and RIP3 by siRNA protected against DHQ3 but not 17-DR induced cell death. These results were confirmed by electron microscopy. DHQ3 and 17-DR induced the degradation of Hsp90 client proteins, and they showed strong antitumor effects in MDA-MB-231 cell-xenografted nude mice.

**Conclusions:**

These findings supported that DHQ3 and 17-DR induce different forms of death in some cancer cell line via activation of different pathways. All of the results provided evidence for its anti-tumorigentic action with low hepatotoxicity in vivo, making them promising anti-breast cancer agents.

## Background

Breast cancer is the most common life-threatening carcinoma, affecting women worldwide. Although considerable progress in treatment development has been made over the past decades, the incidence of breast cancer has been rising sharply in developing countries [1]. Therefore, there is a requirement to develop novel therapeutic agents to treat breast cancer.

Hsp90 and its co-chaperones stabilize various growth factor receptors, signal transduction proteins such as PI3K and AKT, and mutant proteins in cancerous cells [2, 3]. Inhibiting Hsp90 in these cells selectively induces the degradation of over-expressed or mutated proteins, thereby curbing cancer cell growth. Increased expression levels of Hsps are common in human breast cancers, making them potential selective targets [4]. Small molecular Hsp90 inhibitors have been developed and demonstrated promising anti-cancer profiles [5]. Geldanamycin (GA) was identified as the first natural inhibitor of Hsp90. However due to its poor aqueous solubility and its toxicity, GA has not been considered in clinical trials [6, 7]. Therefore, new GA derivatives with improved pharmacological profiles are needed. DHQ3 and 17-demethoxy-reblastatin (17-DR), two non-quinone GA analogs, were obtained from a genetically modified strain of *Streptomyces hygroscopicus* JCM442 and their structures have been determined [8, 9]. The phenolic structure effectively improved the water solubility as compared to the benzoquinone structure. Their ATPase inhibition activity has been proved, but their anti-tumor proliferative activities remain unclear.
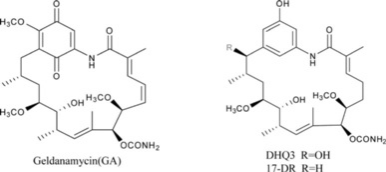


Our previous work showed that the GA analogs could induce cell death in breast cancer cells [10] and human hepatocellular carcinoma cells [11]. Historically, cell death has been classified into distinct forms, including apoptosis, necrosis and autophagy. Caspase activation plays an essential role in the apoptotic process [12, 13]. In the absence of caspase activation, a regulated cellular necrosis, called necroptosis, prevails [14–16]. In the necroptosis process, receptor-interacting protein (RIP) kinase family works together with death receptor proteins to regulate cell death. Recent studies have revealed that RIP3 kinase functions with RIP1 at the intersections of apoptosis, necroptosis, and cell survival [17]. RIP3 is a key determinant of necroptosis [18], the serine phosphorylation is required for the interaction of RIP3 with its substrate mixed lineage kinase domain-like protein (MLKL) [19]. RIP1 and RIP3 form the ‘necrosome’ and subsequently phosphorylate MLKL, causing necroptosis in various cell types [20–22]. Emerging evidence suggests that CaMKII [23], Hsp90 and co-chaperone CDC37 [5] are required for RIP3 activation during necroptosis. In addition, necroptosis can be specifically inhibited by necrostain-1 (Nec-1), a small molecule targeting the death domain kinase RIP1 [14].

Herein, we demonstrated that DHQ3 induces necroptosis in MDA-MB-231 cells through effects on the RIP1-RIP3-MLKL cascade, while 17-DR induces caspase-dependent apoptosis. However, these results were not observed in other cancer cell lines. These two new compounds showed highly effective antitumor activity in vitro and in vivo against breast cancer, providing a foundation for targeted breast cancer therapies.

## Methods

### Reagents and antibodies

DHQ3 and 17-DR were obtained as described previously. They were dissolved in dimethyl sulfoxide (DMSO, Biosharp, Hefei, China) and stored at −20 °C. MG132, Nec-1, DAPI (4,6-diamidino-2-phenylindole), and 3-(4,5-Dimethylthiazol-2-yl)-2,5-diphenyltetrazolium bromide (MTT) were purchased from Sigma-Aldrich (St. Louis, MO, USA). PI assay kits were purchased from Beyotime Institute of Biotechnology (Wuhan, China) and the Annexin V FITC/PI apoptosis detection kit was purchased from Nanjin KeyGen Biotech (Nanjing, China). The ATP Assay kit was purchased from Merck KGaA (Darmstadt, Germany). Lipofectamine 2000 was purchased from Invitrogen (USA). The following antibodies were used: anti-Mcl-1, anti-PARP, anti-RIP1, anti-RIP3 (Santa Cruz Biotechnology, Santa Cruz, CA, USA); anti-Bcl-2, anti-Bax, anti-HIF1a, anti-CDK4, anti-Her2, anti-EGFR (Proteintech, Chicago, IL, USA); anti-Hsp70, anti-Hsp90, anti-Akt (Cell Signaling Technology, Beverly, Massachusetts, USA); anti-caspase 3 and anti-caspase 8 (ENZO, Switzerland); anti-C-Raf (Abcam, Cambridge, MA, USA); and anti-β-actin (BioSharp, Hefei, China).

### Cell lines and cell culture

The human breast cancer cell lines MDA-MB-231, MCF-7 and T-47D, human hepatocellular carcinoma (HCC) cell lines HepG2 and SMMC7721, human nasopharyngeal carcinoma cell lines HNE1 and CNE-2Z, human gastric cancer cell line SGC7901, human endometrial cancer cell line ISK, and human non-small cell lung cancer cell line A549 were bought from Shanghai Cell Bank (Shanghai, China). The human colon carcinoma SW480 was obtained from the American Type Culture Collection (Manassas, VA, USA). Cells were obtained, frozen and cultured in our laboratory. The cells were maintained in Dulbecco’s modified Eagle’s medium (DMEM) (Gibco) or Roswell Park Memorial Institute (RPMI)-1640 medium (Gibco), supplemented with 10 % fetal bovine serum (FBS), 100 U/mL penicillin, and 100 μg/mL streptomycin. Cells were grown in an atmosphere containing 5%CO_2_ at 37 °C.

### Cell viability assay

Cell were plated at a density of 6 × 10^3^ cells/well in a 96-well plate for 24 h, and then treated with different concentrations of analogs. At time points, 15 μL MTT (5 mg/mL in phosphate-buffered saline, PBS) was added to the wells and the plates were incubated further for 4 h at 37 °C. Then, the solution was removed and replaced with 150 μL of DMSO and the absorbance was measured at 490 nm using a plate reader.

### Colony formation assay

Cells were cultured in 6-well plates (6000 cells/well) overnight. The medium was then exchanged with fresh medium containing DHQ3 or 17-DR. The plates were incubated under cell culture conditions for another 5 days, at which point the medium was removed and the cells were washed twice with PBS and fixed with paraformaldehyde for 10 min at – 20 °C. The colonies were stained with 2 % crystal violet for 10 min, washed with double-distilled water and dried at room temperature, before being counted.

### Flow cytometry

Cells (1.2 × 10^5^cells/well) were seeded in 12-well plates and treated with the analogs. After treatment for 24 h, cells were collected and mixed with 50 μg/mL propidium iodide (PI). Cells permeable to PI were counted by Accuri C6 flow cytometry (BD Biosciences, State of New Jersey, U.S.A). To further analyze cell death, Annexin-V FITC/PI staining was performed on cells treated using the method just described, according to the manufacturer’s instructions.

### DAPI staining

Cells were plated in 6-well plates (1.2 × 10^5^cells/well). Following treatment with DHQ3 or 17-DR for 24 h, the cells were fixed with paraformaldehyde for 10 min at 4 °C, washed twice with ice-cold PBS and incubated with 100 μL of DAPI solution (2 μg/mL) for 5 min in the dark. After washing three times with PBS, the cells were observed using an IX71 fluorescence microscope (Olympus, Tokyo, Japan). Cells death was characterized by chromatin condensation and nuclear fragmentation.

### Measurement of intracellular ATP

Cells (1.2 × 10^5^ cells/well) were seeded in a 6-well plate for 24 h prior to incubation with diverse concentrations of analogs for 5 h. To measure intracellular ATP levels, we used a luminometric-based ATP Assay kit according to the manufacturer^’^s protocol. The signal was measured by a Luminoskan luminometer (Thermo Scientific, Atlanta, GA, USA). The ATP concentration with each treatment was calculated as a percentage of that of the control.

### Evaluation of cell death form by electron microscopy

Cultured cells were washed and fixed with 2 % paraformaldehyde and 3 % glutaraldehyde in 0.1 M PBS (pH 7.4) at 4 °C. Then, the cells were post-fixed with 1 % osmium tetroxide for 1.5 h, washed once and treated with 3 % aqueous uranyl acetone, before being dehydrated with a graded series of ethanol and acetone and embedded in Araldite. Thin sections were cut using a Reichert ultramicrotome (Leica, Wetzlar, Germany), post-stained with 0.3 % lead citrate, and examined by TEM (Olympus JEOL, Peabody, MA, USA).

### Western blot analysis

Cells were harvested and homogenized in RIPA lysis buffer for 30 min on ice, the lysates were centrifuged at 12,000 × *g* for 30 min at 4 °C. A bicinchoninic acid (BCA) assay was used to detected protein concentrations. Equal amounts of total protein were separated by sodium dodecyl sulfate-polyacrylamide gel electrophoresis (SDS-PAGE) and transferred to polyvinylidene fluoride (PVDF) membranes. After the membranes were blocked with 5 % skim milk in PBS with 0.1 % Tween20 for 4 h, they were incubated overnight at 4 °C with primary antibodies, followed by incubation with the corresponding secondary antibodies. The membranes were imaged with gel imaging equipment (Bio-Rad, USA). β-actin was used as a loading control.

### Immunofluorescence

Cells in 12-well plates (1.2 × 10^5^ cells/well) were cultured to reach exponential growth prior to being treated with varying concentrations of analogs for 24 h. After treatment, cells were fixed with paraformaldehyde for 15 min, permeabilized for 10 min in 0.2 % Triton X-100, and incubated for 2 h in blocking buffer (5 % BSA in PBS). Next, cells were incubated with RIP1 or RIP3 antibody overnight at 4 °C, and visualized with FITC-conjugated Goat Anti-Rabbit IgG. The nuclei were stained by incubating cells with 2 μg/mL DAPI in PBS and then washed extensively with PBS. Images were obtained by fluorescence microscopy.

### Small interfering RNA transfection

The RIP1 and RIP3 siRNAs were obtained from GenePharma (China). The siRNA were transiently transfected into MDA-MB-231 cells in 6-well plates using 10 μL Lipofectamine 2000 reagent (Invitrogen, USA) according to the manufacturer’s protocol. After 48 h of transfection, the cells were collected for immunoblot analysis as described earlier. The sequences of siRNA used for experiments of human RIP1 siRNA and RIP3 siRNA were as follows: Negative control sense, 5’-UUC UCC GAA CGU GUC ACG UTT-3’and antisense, 5’-ACG UGA CAC GUU CGG AGA ATT-3’; Positive control sense, 5’-UGA CCU CAA CUA CAU GGU UTT-3’ and antisense, 5’-CUU GAG GCU GUU GUC AUA CTT-3’; RIP1-homo-1980 sense, CCUUCUGAGCAGCUUGAUUTT and antisense, AAUCAAGCUGCUCAGAAGGTT; RIP3-homo-966 sense, CCGGCUUAGAAGGACUGAATT and antisense, UUCAGUCCUUCUAAGCCGGTT.

### Colorimetric determination of ATPase activity

The assay was performed as previously described with slight modifications [24, 25]. To prepare the malachite green reagent, and then, either analog or DMSO control was added. The plate was incubated for 2.5 h at 37 °C. Next, 80 μL of malachite green reagent was added to each well, the plate was shaken and 10 μL of 34 % sodium citrate was added. The samples were mixed thoroughly for 10–20 min before measuring OD_650_ using a microplate reader (Synergy HT, BioTek, Vermont, U.S.A).

### In vivo experiments

To evaluate if antitumor effects delivered by DHQ3 and 17-DR, we used the female nude mice (4- to 6-weeks old) to perform experiments, they were obtained from the animal experimental center of Beijing vitalriver. MDA-MB-231 cells (3×10^6^ cells per animal) were injected subcutaneously to induce tumor formation. Next, 20 mice that developed tumors over 100 mm ^3^ were randomly divided into five groups (5 mice per group), 0.2 ml of DMSO, 17-DR (25 mg/kg), DHQ3 (50 mg/kg), DDP (3 mg/ kg) were intraperitoneally injected every 3 days, and body weight was monitored before each injection. Tumor volume was measured as length x width^2^/2. After treatment 21 days with drugs, the solid tumors were removed and stored in 4 % formalin solution, cut into small pieces that were stained with hematoxylin and eosin (H&E).

### Statistical analysis

Values are expressed as the means ± SEM of three experiments. SPSS v.16.0 software (SPSS Inc., Chicago, IL, USA) was used for data analysis. Comparisons between two groups were analyzed using the two-tailed Student’s *t*-test. A p-value < 0.05 is considered statistically significant.

## Results

### Inhibition of MDA-MB-231 cell proliferation with varying concentrations of DHQ3 and 17-DR

**Fig. 1:**
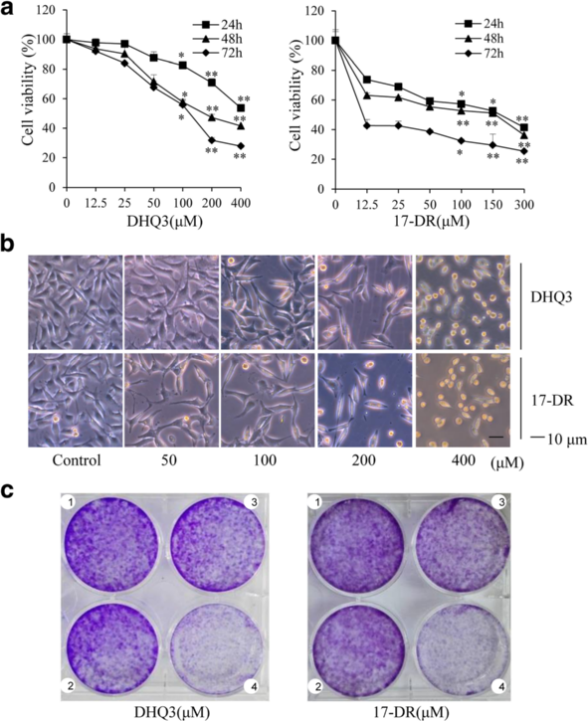
Inhibitory effects of DHQ3 and 17-DR on MDA-MB-231 cells. **a** MDA-MB-231 cells were treated with various concentrations of analogs for 24, 48 or 72 h. Cell viability was measured by MTT assay. *p < 0.01, **p < 0.001. **b** Cells were treated with analogs for 24 h and the morphology was examined by light microscopy with differential interference contrast optics. **c** The colony-forming capability of MDA-MB-231 cells was measured using a colony formation assay after treatment with various concentrations of analogs for 5 days

To investigate the effects of DHQ3 and 17-DR on breast cancer cells, MDA-MB-231 cells were treated with different concentrations of DHQ3 and 17-DR for 24, 48 or 72 h. As determined by MTT assays, DHQ3 and 17-DR significantly inhibited MDA-MB-231 cell growth (Fig. 1a and b), the cell viability rates were gradually reduced with the increase of analogs concentration (*p < 0.01*). Furthermore, we observed that DHQ3 and 17-DR could inhibit MDA-MB-231 cells colony formation by increasing concentrations (Fig. 1c).

### DHQ3 and 17-DR induce different forms of cell death in MDA-MB-231 cells

**Fig. 2:**
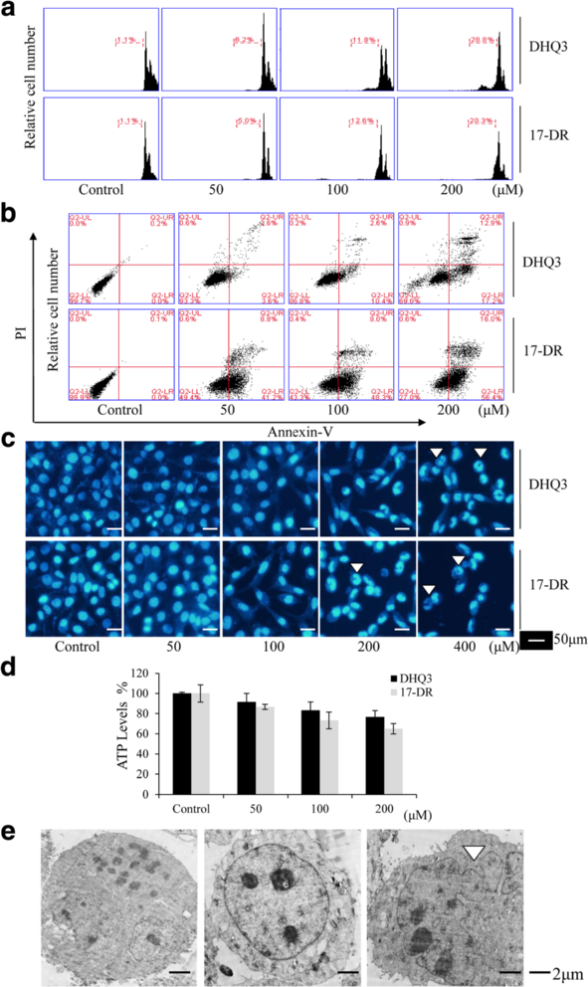
DHQ3 and 17-DR induced various forms of death in MDA-MB-231 breast cancer cells. **a** Flow cytometric analysis of cell death after treatment with DHQ3 or 17-DR for 24 h using PI staining. **b** Cells treated with analogs for 24 h were analyzed using an Annexin V-FITC/PI assay. **c** Cells were treated with the indicated concentrations of analogs for 24 h, subjected to DAPI staining (showing nucleus) and visualized by fluorescence microscopy. White arrowheads indicate dead cells. **d** Reduction in ATP production after analogs treatment. Cellular levels of ATP were measured after 5 h. **e** DHQ3 induced necroptosis, while 17-DR induced apoptosis in MDA-MB-231 cells. Electron microscopy of cells treated for 24 h with DMSO, 200 μM DHQ3 or 200 μM 17-DR. White arrowheads denote chromatin pyknosis in cells treated with 17-DR

Similarly, MDA-MB-231 cells were treated with DHQ3 or 17-DR for 24 h and then flow cytometry was used to detect dead cells. As expected, PI-staining indicated an increase in the ratio of dead cells with increasing analog concentrations (Fig. 2a), suggesting that both analogs induce cell death in MDA-MB-231 breast cancer cells. Annexin-V FITC/PI staining gave similar results, with 17-DR treatment, we could see a large number of early stage apoptotic cells were in the AV + PI- quadrant (Fig. 2b). With analog treatment, DAPI-stained cells exhibited condensed and fragmented nuclei, which is indicative of cell death (Fig. 2c). Furthermore, it has been debated whether the function of Hsp90 is ATP dependent. It was found that cellular ATP levels were reduced with DHQ3 and 17-DR treatment (Fig. 2d), suggesting that the analogs induced cell death by decreasing ATP levels. To further define the types of cell death obtained with analogs treatment, we observed cells under an electron microscope (Fig. 2e). DHQ3 induced typical nuclear fragmentation, loss of plasma membrane integrity, and organelle (especially mitochondrial) swelling. At the same time, cells treated with 17-DR showed typical characteristics of apoptosis: nucleus concentrating, condensation and margination of nuclear chromatin, and nuclear membrane and plasma membrane and organelles intact.

### 17-DR-induced apoptosis is caspase-dependent

**Fig. 3:**
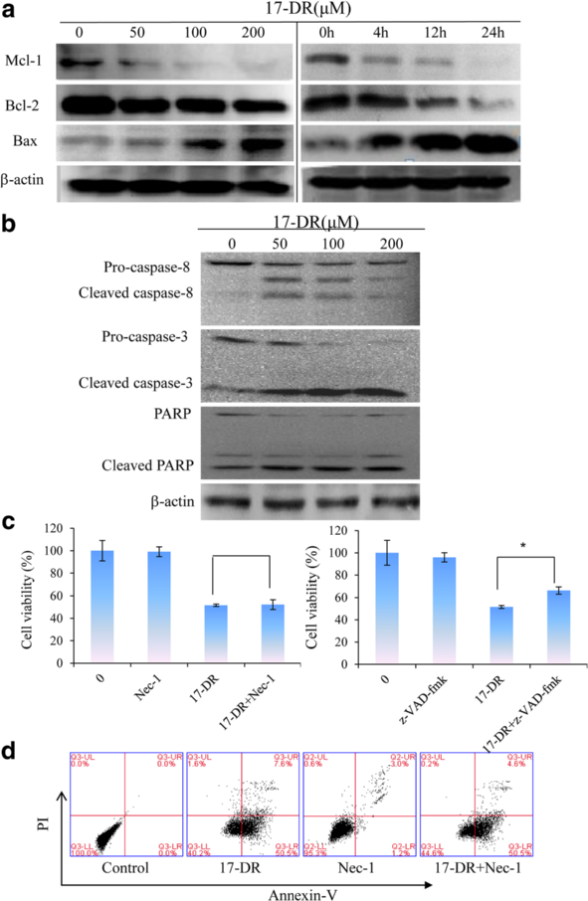
17-DR induced apoptosis in MDA-MB-231 cells that is dependent of caspase activity. **a** Whole-cell lysates from the MDA-MB-231 cells treated with different concentrations of 17-DR, or treated with 200 μM 17-DR for different lengths of time, were subjected to western blot analysis. **b** The expression of caspase-3, −8 and PARP. 17-DR increased their activation at 24 h. **c** Analysis of cell viability by MTT assay, following treatment with 17-DR with or without 1 h pre-treatment with Nec-1 or z-VAD-fmk. *P < 0.05. **d** Annexin V-FITC/PI analysis following treatment with 17-DR alone or with Nec-1 pre-treatment

To verify whether the 17-DR-induced cell death observed in MDA-MB-231 cells was a result of apoptosis, western blot analyses were conducted to measure the levels of apoptosis proteins. Apoptosis was induced in breast cancer cells by 17-DR as confirmed by the down-regulation of Mcl-1 and Bcl-2, and up-regulation of Bax protein levels (Fig. 3a). Further, we performed immunoblotting to detect caspase activation. The cleaved products of caspase-3, caspase-8 and PARP were detected (Fig. 3b), indicating that 17-DR-induced cell death was apoptotic. Furthermore, we pre-treated cells with the necroptosis inhibitor, Nec-1 or the caspase inhibitor, z-VAD-fmk. The effects of 17-DR were decreased by treatment with the apoptosis inhibitor z-VAD-fmk (Fig. 3c), the cell viability was (51.59 ± 1.27) % when 17-DR used alone, increased to (66.34 ± 3.25) % in conjunction with z-VAD-fmk (*p < 0.01*), but not by Nec-1 (Fig. 3c and d), indicating a caspase-independent killing mechanism.

### Hsp90 inhibitor DHQ3 induces necroptosis in breast cancer MDA-MB-231 cells

**Fig. 4:**
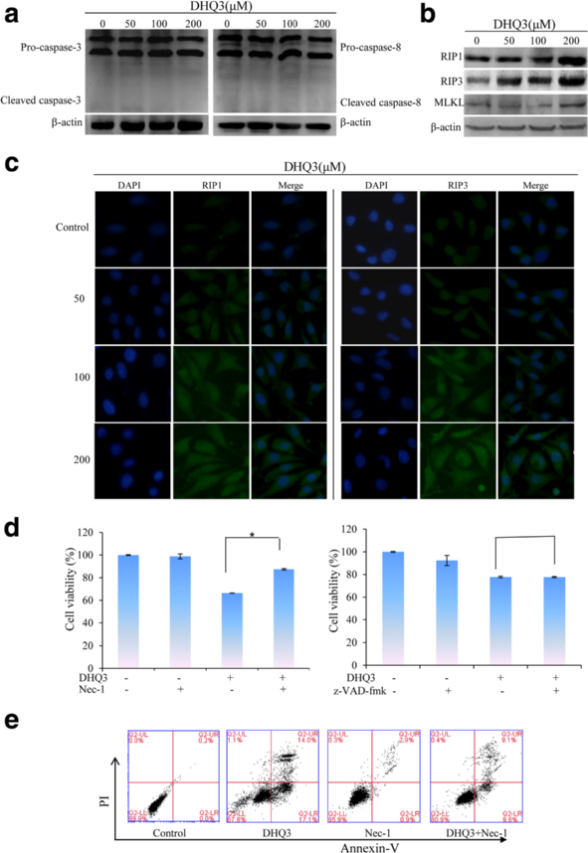
DHQ3 induced necroptosis in MDA-MB-231 cells. **a** Cells were treated with DHQ3 for 24 h. Cell lysates were prepared and examined using western blot analysis. **b** The expression of necroptosis-related proteins was analyzed by western blot. **c** MDA-MB-231 cells were incubated with DHQ3 for 24 h and treated with primary antibodies overnight at 4 °C. The localization of the target proteins was assessed by immunofluorescence staining. Nuclei were stained with DAPI. Scale bar is 50 μm. **d** Cells incubated with DHQ3 (200 μM) for 24 h, with or without 1 h pre-treatment with Nec-1 or z-VAD-fmk, were analyzed by the MTT assay. *P < 0.05. **e** MDA-MB-231 cells treated with DMSO, Nec-1 (20 μM), DHQ3 (200 μM) alone or with Nec-1 pre-treatment, were analyzed by Annexin V-FITC/PI assay

Unlike 17-DR treated cells, cleaved products of caspase-3 and caspase-8 were nearly undetected in DHQ3 treated cells (Fig. 4a). Next, we found that DHQ3 up-regulated expression of RIP1, RIP3 and MLKL (Fig. 4b). In addition, DHQ3 significantly promoted the nuclear translocation of RIP1 and RIP3 (Fig. 4c), they showed DHQ3 increased the expression of RIP1 and RIP3 on the cytoplasm. The viability of DHQ3-treated cells was rescued with Nec-1 pre-treatment (Fig. 4d), there was an increase [(87.39 ± 0.75) %] in conjunction with Nec-1 (*p < 0.01*) compared to DHQ3-treated alone [(66.32 ± 0.39) %]. As proven by Annexin-V FITC/PI staining (Fig. 4e), DHQ3/Nec-1 induced less cell death than did DHQ3 alone. Nec-1 significantly protected the cells from DHQ3-induced, reducing right quadrant populations from 31.1 to 18.7 %. On the other hand, z-VAD-fmk did not protect against DHQ3-induced cell death (Fig. 4d). These results demonstrated that DHQ3 induced necroptosis in breast cancer MDA-MB-231 cells.

### Knockdown of RIP with siRNA protected MDA-MB-231 cells against DHQ3-induced necroptosis

**Fig. 5:**
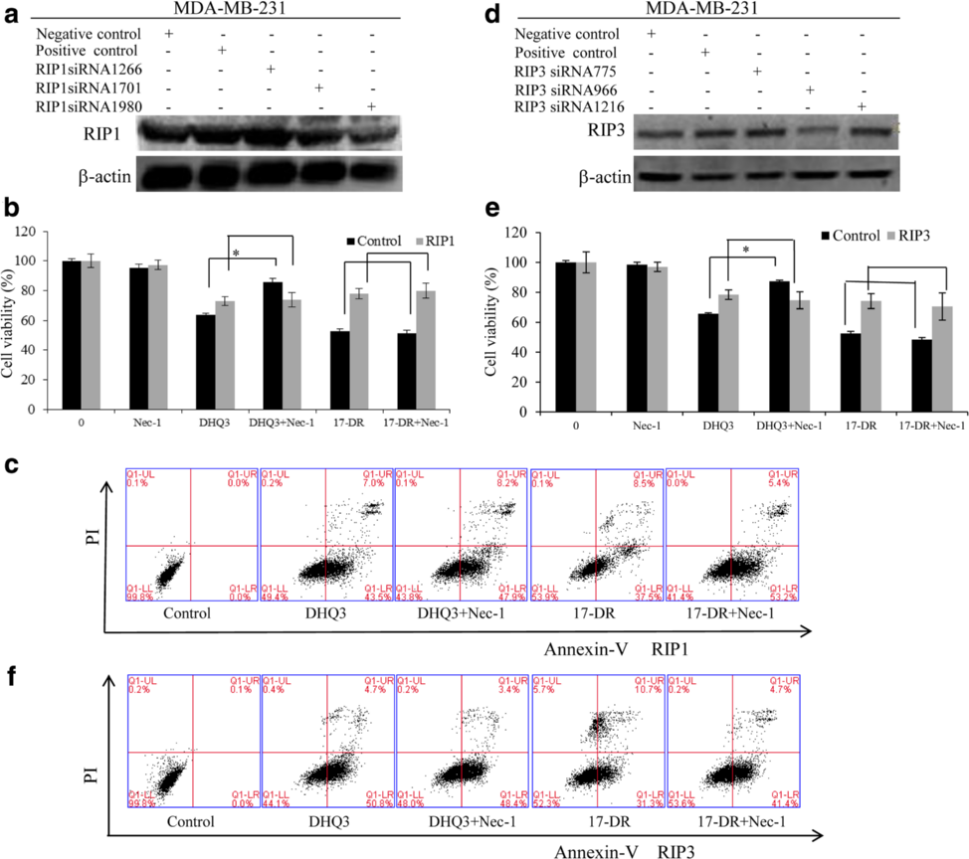
Knockdown of RIPs with siRNA protected against DHQ3-induced necroptosis in MDA-MB-231 cells. **a**, **d** MDA-MB-231 cells were transfected with control, or RIP1 or RIP3 siRNA, and after 48 h, whole-cell lysates were subjected to western blot analysis. **b**, **e** After 24 h transfection with RIP1/RIP3 siRNA, cells were treated with 200 μM of analogs for another 24 h, with or without 1 h pre-treatment with Nec-1 and z-VAD-fmk. Cell viability was measured by MTT assay. *P < 0.05. **c**, **f** Similarly to b, MDA-MB-231 cells were analyzed by Annexin V-FITC/PI assay

To characterize the mechanisms underlying sensitization to the analogs, we investigated the effect of RIP1 and RIP3 knockdowns on the signaling pathway in MDA-MB-231 cells. Results shown in Fig. 5 demonstrated that silencing RIP1 and RIP3 expression prevented DHQ3 from activating cell death, and caused the loss of Nec-1’s protective effect. However, silencing RIP1 expression (Fig. 5a) did not affect 17-DR-induced cell death, as shown in the MTT and Annexin-V FITC/PI results (Fig. 5b and c). In the normal, the viability was (87.39 ± 0.70) % when combined with Nec-1, compared (65.75 ± 0.60) % that treated DHQ3 alone; after the silence of RIP1, the viability was (74.67 ± 5.61) % in conjunction with Nec-1, compared (78.42 ± 3.22) % that treated DHQ3 alone. The same results occurred when RIP3 was silencing (Fig. 5d). The cell viability were (63.53 ± 1.44) % and (72.95 ± 2.91) % in the before and after processing RIP3 silence when treated DHQ3 alone, and changed to (85.84 ± 2.33) % and (73.94 ± 4.76) % when combined with Nec-1 (Fig. 5e). Consistent with MTT results, the Annexin-V FITC/PI results approved again, Nec-1 lost its protection for DHQ3 (Fig. 5f). Altogether, these data demonstrated that in MDA-MB-231 cells DHQ3 induced necroptosis, which can be mimicked by down-regulation of RIP1 and RIP3.

### DHQ3 and 17-DR down-regulate Hsp90 client proteins in breast cancer MDA-MB-231 cells

**Fig. 6:**
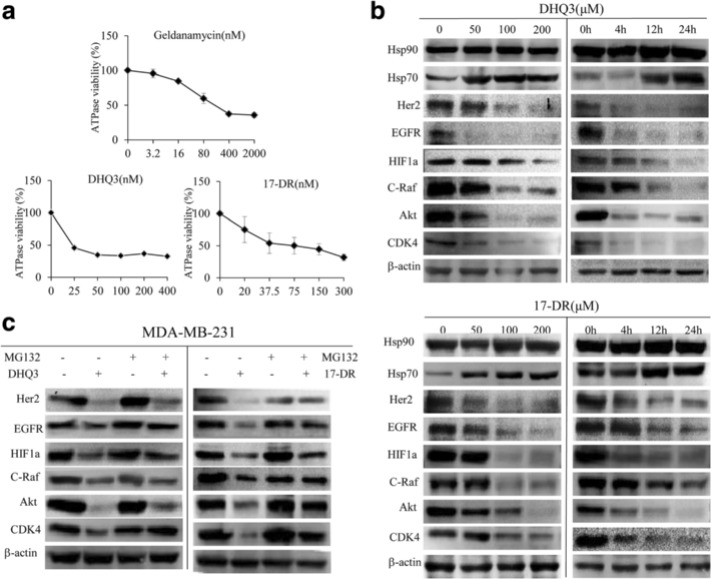
DHQ3 and 17-DR induced the degradation of Hsp90 client proteins. **a** Hsp90 ATPase activity at different concentrations of GA, DHQ3 and 17-DR were tested by the malachite green-phosphate and ammonium molybdate method. At the given times, the reaction was stopped and the OD_650_ value measured. **b** Cells were treated with analogs at different concentrations or different lengths of time, the Hsp90 client proteins were assessed by western blot. **c** MDA-MB-231 cells were pretreated with 10 μM MG132 for 4 h, with or without a subsequent 24 h incubation with 200 μM DHQ3 or 200 μM 17-DR. Whole-cell lysates were subjected to western blot analysis

Hsp90 inhibitors have been established as a method of targeting Hsp90 client onco-proteins [26–28] and promoting apoptosis of tumor cells [29]. The isolated analogs were tested for their ability to inhibit yeast Hsp90 activity using the malachite green ATPase assay. Using the optimized reaction conditions, DHQ3 and 17-DR presented stronger ATPase inhibition activity compared to the original Hsp90 inhibitor, GA (Fig. 6a). To confirm the regulation of Hsp90 by DHQ3 and 17-DR, the expression levels of its client proteins were examined, including Akt, Her2, CDK4, C-Raf, HIF1α and EGFR [3, 30]. Western blot analysis demonstrated that these classical client proteins were significantly down-regulated in the presence of DHQ3 and 17-DR (Fig. 6b). Several reports have suggested that the decrease in expression levels of Hsp90 client proteins is due to their degradation by the proteasome. Indeed, a 1 h pre-treatment with the proteasome inhibitor MG132 was sufficient to partly restore the expression level of client proteins in cells incubated with DHQ3 or 17-DR (Fig. 6c).

### In vivo antitumor efficacy of 17-DR or DHQ3

**Fig. 7:**
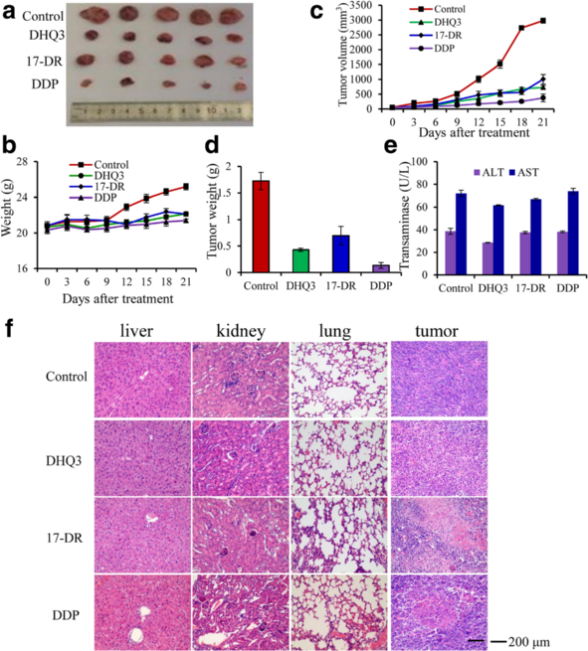
DHQ3 and 17-DR significantly delays MDA-MB-231 tumor growth. **a** Representative tumors from each treatment group. **b** Body weight of the mice. **c** Tumor volume of the mice. **d** Tumor weights after DHQ3, 17-DR and DDP treatment. **e** In vivo hepatotoxicity evaluation of DHQ3 and 17-DR in nude mice. The AST and ALT were determined by Assay Kit. The blood serum samples were treated according to the manufacturer’s instruction. And the AST and ALT activities are expressed as U/L. **f** H&E-stained sections of the tumor, liver, lung and kidney from the mice after treatment

To test whether 17-DR or DHQ3 show any antitumor effects in vivo, MDA-MB-231 cells were xenografted into nude mice, and then the tumor-bearing animals were treated with 17-DR (25 mg/kg/3days) or DHQ3 (50 mg/kg/3days) for 21 days by intraperitoneal injection. We observed that 17-DR or DHQ3 prevented tumor growth (Fig. 7a) compared with the control. Obvious body weight loss was observed after treatment with DHQ3 and 17-DR (Fig. 7b). When each animal was considered individually, the incidence of mice progressing with a tumor volume of 500 mm^3^ was significantly diminished by day 15 in DHQ3/17-DR-treated animals compared with controls (Fig. 7c). Consistent with body weight data, 17-DR- or DHQ3-treated groups exhibited obvious decrease in tumor weight (Fig. 7d). Aspartate aminotransferase (AST) and alanine-aminotransferase (ALT) are commonly used as biomarkers for the evaluation of hepatotoxicity [31], after the treatment of 17-DR or DHQ3, the levels of AST and ALT were analyzed using an activity assays, showing that 17-DR and DHQ3 had nearly no effect on the AST and ALT compared with those of the control group, but obviously lower than those of the DDP-treated group (Fig. 7e). H&E staining of the organs such as the liver, lung and kidney demonstrate that there was no serious damage (Fig. 7f). All of the results provided evidence for its anti-tumorigenic action with low hepatotoxicity in vivo.

### DHQ3 and 17-DR do not induce different forms of death in other cancer cell lines

**Fig. 8:**
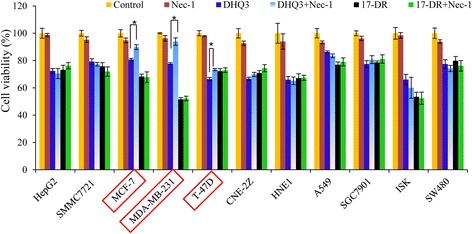
MTT results of DHQ3 and 17-DR on ten human cancer cell lines. HepG2, SMMC7721, MCF-7, MDA-MB-231, T-47D,CNE-2Z, HNE1, A549, SGC7901, ISK, SW480 were exposed to 200 μM analogs as in previous experiments with MDA-MB-231 cells for 24 h, with or without pre-treatment with Nec-1. The cell survival rates were assessed using MTT assays. *P < 0.05

Based on these interesting data, a series of cancer cell lines were treated with the same concentrations of DHQ3 or 17-DR, as well as pre-treated with Nec-1, as in the experiments with MDA-MB-231 cells (Fig. 8). Under the conditions used, the same phenomenon of different forms of cell death with different analogs was not observed in these other cancer cell lines. Remarkably, combined with Nec-1 rescued MCF-7, MDA-MB-231, T-47D cells to DHQ3. However, Nec-1 did not rescue HepG2, SMMC7721, CNE-2Z, HNE1, A549, SGC7901, ISK and SW480 cells from DHQ3-induced cell death at all.

## Discussion

The MDA-MB-231 cell line is a type of triple-negative breast cancers (TNBC), which has been tested negative for estrogen receptors, progesterone receptors and HER2 [32]. Relatively few drugs work against TNBC, and there is a lack of standardized medication guidelines for TNBC. The expression of Hsp90 in tumor cells is 2–10 times higher than in normal cells, therefore, Hsp90 inhibitors could show selectivity towards tumor cells, yielding specific antitumor effects [7, 33]. In study, DHQ3 and 17-DR presented potent antitumor activity for TNBC in vitro and in vivo.

On the basis of the strong ties between Hsp90 and carcinogenesis, a significant proportion of the work on Hsp90 inhibitors have been committed to developing derivatives of GA, including 17-AAG and 17-ABAG [34–37]. However, there seems to be a limit to further development of most of these inhibitors. Therefore, there is a need for further analysis of analogs. Our findings indicated that the new non-quinone GA analogs DHQ3 and 17-DR have high activity against a series of cancer cells, especially MDA-MB-231 cells. As Hsp90 inhibitors, they were tested for the ability to inhibit yeast Hsp90 activity using a malachite green ATPase assay, and displayed stronger inhibition of ATPase activity than GA. Furthermore, down-regulated the levels of Hsp90 client proteins, and the degradations of these client proteins were blocked by treatment with the proteasome inhibitor MG132. More importantly, the result of this study showed that DHQ3 and 17-DR have potent inhibitory effects on MDA-MB-231 breast cancer cell tumor growth, and showed low hepatotoxicity in vivo and no serious side effects.

Interestingly, DHQ3-induced necroptosis is dependent on RIP1 and RIP3, a classical mode of necroptosis, while 17-DR-treated cells display the characteristic changes of apoptosis. We found that 17-DR regulates apoptosis-related proteins, and that it induces apoptosis of MDA-MB-231 cells in a caspase-3, −8 dependent manner. Our data also demonstrated that treatment with DHQ3 resulted in significant induction of necroptosis associated with expression of RIP. Recent research has underlined RIP3 as a key protein regulating the switch between TNF-induced necroptosis and survival, and indicated that the complex containing RIP3 could function as a “necrosome” which differ from other complexes that induce apoptosis or NF-kB activation [17, 18, 38]. The state of RIP determines whether it functions as a molecule that promotes or inhibits cell death [39]. Our work showed that DHQ3 treatment markedly increased the level of RIP1 and RIP3 in MDA-MB-231.

It is puzzling that the two analogs with similar structure would guide MDA-MB-231 cells towards two different cell death programs. In our siRNA studies, we clearly demonstrated the importance of RIP1 and RIP3 in the death process. The data indicate that knockdown of RIP1 and RIP3 expression inhibit DHQ3-induced necroptosis, and cause the loss of protective effect of Nec-1, but has no effect on 17-DR-induced apoptosis. Furthermore, it has been revealed that GA accelerates the molecular switch from necroptosis to apoptosis via RIP1 down-regulation in TNF-stimulated L929 cells [40]. As described above, RIP is one of the client protein of Hsp90, therefore we hypothesize that the two Hsp90 inhibitors induce different forms of death by having slightly different effects on RIP. This is of importance and merits further investigation. Further, in-depth testing is required to determine the efficacy.

## Conclusions

In summary, the two new non-benzoquinone GA analogs, DHQ3 and 17-DR, inhibited the proliferation of MDA-MB-231 cells by decreasing the expression of Hsp90 client proteins. They have potent inhibitory effects on MDA-MB-231 breast cancer cell tumor growth with low hepatotoxicity. Importantly, our observations revealed that they triggered different forms of death in MDA-MB-231 cells. DHQ3 functions as an Hsp90 inhibitor and a necroptosis inducer, while 17-DR induces caspase-dependent apoptosis.

## Abbreviations

17-DR: 17-demethoxy-reblastatin
Akt: Serine/threonine kinase
ALT: Alanine-aminotransferase
AST: Aspartate aminotransferase
BSA: Bovine serum albumin
CDK4: Cyclin dependent kinase 4
C-Raf: Raf proto-oncogene serine/threonine-protein kinase
DAPI: 4,6-diamidino-2-phenylindole
DMEM: Dulbecco’s modified Eagle’s medium
DMSO: Dimethyl sulfoxide
EGFR: Epidermal growth factor receptor
FBS: Fetal bovine serum
GA: Geldanamycin
HCC: Human hepatocellular carcinoma
Her2: Human epidermal growth factor receptor 2
HIF1a: Hypoxia-inducible factor
HSPs: Heat shock proteins
MLKL: Mixed lineage kinase domain-like protein
MTT: 3-(4,5-Dimethylthiazol-2-yl)-2,5-diphenyltetrazolium bromide
Nec-1: Necrostatin-1
PBS: Phosphate-buffered saline
PI: Propidium iodide
PVDF: Polyvinylidene fluoride
RIP: Receptor-interacting protein
RPMI-1640: Roswell park memorial institute-1640 medium
SDS-PAGE: Sodium dodecyl sulfate-polyacrylamide gel electrophoresis
TNBC: Triple-negative breast cancers
